# Minimally invasive and open gastrectomy for gastric cancer: A protocol for systematic review and network meta-analysis

**DOI:** 10.1097/MD.0000000000013419

**Published:** 2018-11-30

**Authors:** Xixiong Wang, Zhiqiang Li, Meizhu Chen, Chenming Wu, Yexiang Fu

**Affiliations:** a Department of Surgical Oncology, Boao Evergrande International Hospital, Qionghai; b Department of Gastrointestinal Surgery, Sanya People's Hospital, Sanya, China.

**Keywords:** gastrectomy, gastric cancer, laparoscopic, network meta-analysis, robotic

## Abstract

**Background:**

The aim of this study is to find the better treatment for gastric cancer by comparing robotic gastrectomy, laparoscopic gastrectomy, and open gastrectomy using Bayesian network meta-analysis.

**Methods:**

We will search PubMed, Embase, and the Cochrane Library for eligible studies published before 1 September 2018. There will be no language restrictions. Randomized clinical trials that compare robotic gastrectomy, laparoscopic gastrectomy, or open gastrectomy for patients with gastric cancer will be included. The risk of bias of included studies will be assessed by the Cochrane Collaboration's tool for assessing risk of bias in randomized trial. The outcomes of the study include operation time, estimated blood loss, time of ambulation, times to first flatus, time of oral intake, hospitalization, and the occurrence of complication. If sufficient data is collected and adequate clinical homogeneity is established among studies, we will conduct pairwise meta-analyses and Bayesian network meta-analyses for all related outcome measures.

**Ethics and dissemination:**

The study does not involve human subjects and does not need ethical approval and patient consent. The results of the network meta-analysis will be disseminated in a peer-reviewed journal for publication.

## 1 Introduction

Gastric cancer is the third leading cause of cancer death worldwide in 2016.^[1]^ Surgical resection remains the gold standard of treatment for localized gastric cancer.^[2,3]^ Laparoscopic gastrectomy has now been widely accepted for the treatment of early gastric cancer since Kitano et al first reported the use of laparoscopy in distal gastrectomy.^[4,5]^ The safety and feasibility of laparoscopic surgery have also been established in randomized controlled trials (RCTs) and meta-analyses.^[6–8]^

Laparoscopy, however, has some limitations including the restricted device manipulation area, amplification of hand tremor, and 2-dimensional imaging.^[9]^ To overcome the disadvantages of laparoscopy, robotic surgical systems which significantly improve visibility, and manipulation have been introduced.^[10,11]^ Numerous studies have assessed the efficacy and safety of robotic gastrectomy.^[9,12–16]^

Currently, the comparative effectiveness and safety of minimally invasive and open gastrectomy for gastric cancer are still unknown because of the limited number of head-to-head trials. On the other hand, pairwise meta-analyses could only combine direct evidence. Network meta-analyses which could synthesize both the direct evidence and indirect evidence simultaneously is a potential solution to the problems. Therefore, in the present study, we will evaluate the comparative efficacy and safety of robotic gastrectomy, laparoscopic gastrectomy, and open gastrectomy on gastric cancer using a Bayesian network meta-analysis to find the best treatment for gastric cancer.

## 2 Methods

The protocol adheres to preferred reporting items for systematic review and meta-analysis protocol (PRISMA-P) checklist.^[17]^ The network meta-analysis will be conducted in accordance with PRISMA for Network Meta-Analyses guidelines.^[18]^ The study has been registered in PROSPERO (CRD42018108687). The study does not involve human subjects and does not need ethical approval and patient consent.

### 2.1 Eligibility criteria

#### 2.1.1 Participants and interventions

We will include studies focusing on patients with gastric cancer. Comparisons of laparoscopic gastrectomy, robotic gastrectomy, or open gastrectomy will be included. No further restrictions will be made on age, ethnic distribution, and gender.

#### 2.1.2 Outcomes

The outcomes of the study include operation time, estimated blood loss, time of ambulation, times to first flatus, time of oral intake, hospitalization, and the occurrence of complication.

#### 2.1.3 Study design

Study designs of interest will include only published RCTs comparing laparoscopic gastrectomy or robotic gastrectomy or open gastrectomy. There is no language restriction.

### 2.2 Information sources and search strategy

We will search the following databases: PubMed, Embase, and the Cochrane library. All databases will be systematically searched from implementation to 1 September 2018.

Search strategy of PubMed was as follows:

- #1 ((“stomach neoplasms”[MeSH Terms] or (“stomach”[All Fields] and “neoplasms”[All Fields]) or “stomach neoplasms”[All Fields] or (“gastric”[All Fields] and “cancer”[All Fields]) or “gastric cancer”[All Fields])) or “stomach cancer”
- #2 (“robotics”[MeSH Terms] or “robotics”[All Fields] or “robotic”[All Fields]) and (“gastrectomy”[MeSH Terms] or “gastrectomy”[All Fields])
- #3 (“laparoscopy”[MeSH Terms] or “laparoscopy”[All Fields] or “laparoscopic”[All Fields]) and (“gastrectomy”[MeSH Terms] or “gastrectomy”[All Fields])
- #4 #2 or #3
- #5 RCT[Publication Type] or randomized[Title/Abstract] or placebo[Title/Abstract]
- #6 #1 and #4 and #5

### 2.3 Selection process and data management

Two independent reviewers will complete study selection and data management. Reviewers will evaluate the study titles and abstracts to include articles that meet the inclusion criteria. Disagreements will be resolved by discussion.

For studies that meet the eligibility criteria, we will extract data from articles into a standardized form. The data includes characteristics of the study (first author, year, geographical region, number of patients of the laparoscopic group, robotic gastrectomy group, or open gastrectomy group), baseline characteristics of participants (age, final pathology, TNM stage) and outcomes. Moreover, follow-up information and survival data will be collected.

If standard deviations (SDs) or standard errors were not reported for continuous outcomes, we will first calculate effect size in Review Manager version 5.3 based on the reported data such as 95% confidence intervals (CIs) or *P* value. If the effect size cannot be calculated, we will contact the authors to obtain additional data. If there is no response, we will send 2 email reminders to study authors.

### 2.4 Risk of bias

The risk of bias in individual studies will be assessed by 2 independent researchers in accordance with Cochrane Collaboration's tool for assessing risk of bias in randomized trials.^[19]^ Assessment of the risk of bias will be based on following domains: sequence generation, allocation concealment, blinding of participants and researchers, incomplete outcome data, selective reporting, and other bias random.^[19]^ Disagreements will be resolved through discussion.

### 2.5 Data synthesis and statistical analysis

#### 2.5.1 Measures of treatment effects

We will calculate continuous data using the weighted mean difference (WMD) with 95% credible intervals (CrIs) and dichotomous variables using odd ratio (OR) with 95% CrIs. We can interpret CrIs as CIs, and a 2-sided *P* <.05 can be assumed if 95% CrIs do not include 0 at conventional levels of statistical significance.^[20]^ We will assess heterogeneity using chi-square test and I^2^ statistic. Chi-square test with the significance set *P* <.10 or I^2^ >50% indicates statistical heterogeneity.^[21]^

#### 2.5.2 Data analysis

First, we will perform traditional pairwise meta-analyses with random-effects model^[22]^ for every head-to-head comparison involving at least 2 RCTs. Then, we will conduct a network meta-analysis with a Bayesian random-effects model. Apart from pooled WMDs or odds ratios (ORs) with 95% CrIs, relative ranking probability of different types of gastrectomy will be presented through surface under cumulative ranking curve (SUCRA) values. SUCRA values will be expressed as percentages of efficacy or safety of each intervention, and a larger the SUCRA value indicate the better the rank.^[23]^ For each analysis, we will assess the inconsistencies between direct and indirect evidence in the network using node splitting method.^[24]^ Comparison-adjusted funnel plots will be drawn to estimate publication bias in the network meta-analysis.^[25]^ We will draw network plots to present the geometry of the network meta-analyses.

#### 2.5.3 Software

All analyses involved will be performed using R v3.5.0 (gemtc package and rjags package), and Stata version 14.

## 3 Discussion

To date, no network meta-analysis has been carried out to provide comprehensive quantitative synthesis of robotic gastrectomy, laparoscopic gastrectomy, and open gastrectomy. In this systematic review and Bayesian network meta-analysis, we will summarize direct and indirect evidence and evaluate comparative the efficacy and safety of minimally invasive and open gastrectomy for gastric cancer. Our study will rank robotic gastrectomy, laparoscopic gastrectomy, and open gastrectomy for gastric cancer based on the relevant outcomes. The results of this study may be beneficial for patients with gastric cancer, gastrointestinal surgeons and policy-makers.

## Author contributions

Xixiong Wang conceived the concept and designed the study protocol. Zhiqiang Li and Meizhu Chen tested the feasibility of the study. Chenming Wu, Yexiang Fu wrote the manuscript. Xixiong Wang provided methodological advice, polished and revised the manuscript. All authors saw and approved the final version of the paper.

**Conceptualization:** Xixiong Wang.

**Investigation:** Zhiqiang Li, Meizhu Chen.

**Methodology:** Xixiong Wang.

**Writing – original draft:** Chenming Wu, Yexiang Fu.

**Writing – review & editing:** Xixiong Wang.
